# Impact of Different Modules of 21-Gene Assay in Early Breast Cancer Patients

**DOI:** 10.3389/fendo.2021.759338

**Published:** 2021-11-02

**Authors:** Mengdi Chen, Deyue Liu, Weilin Chen, Weiguo Chen, Kunwei Shen, Jiayi Wu, Li Zhu

**Affiliations:** ^1^ Department of General Surgery, Comprehensive Breast Health Center, Ruijin Hospital, Shanghai Jiao Tong University School of Medicine, Shanghai, China; ^2^ Department of Thyroid and Breast Surgery, Shanghai General Hospital, Shanghai Jiao Tong University School, Shanghai, China

**Keywords:** breast cancer, hormone receptor positive, recurrence score, 21-gene assay, adjuvant therapies

## Abstract

**Background:**

The 21-gene assay recurrence score (RS) provides additional information on recurrence risk of breast cancer patients and prediction of chemotherapy benefit. Previous studies that examined the contribution of the individual genes and gene modules of RS were conducted mostly in postmenopausal patients. We aimed to evaluate the gene modules of RS in patients of different ages.

**Methods:**

A total of 1,078 estrogen receptor (ER)-positive and human epidermal growth factor receptor 2 (HER2)-negative breast cancer patients diagnosed between January 2009 and March 2017 from Shanghai Jiao Tong University Breast Cancer Data Base were included. All patients were divided into three subgroups: Group A, ≤40 years and premenopausal (*n* = 97); Group B, >40 years and premenopausal (*n* = 284); Group C, postmenopausal (*n* = 697). The estrogen, proliferation, invasion, and HER2 module scores from RS were used to characterize the respective molecular features. Spearman correlation and analysis of the variance tests were conducted for RS and its constituent modules.

**Results:**

In patients >40 years, RS had a strong negative correlation with its estrogen module (*ρ* = −0.76 and −0.79 in Groups B and C) and a weak positive correlation with its invasion module (*ρ* = 0.29 and 0.25 in Groups B and C). The proliferation module mostly contributed to the variance in young patients (37.3%) while the ER module contributed most in old patients (54.1% and 53.4% in Groups B and C). In the genetic high-risk (RS >25) group, the proliferation module was the leading driver in all patients (*ρ* = 0.38, 0.53, and 0.52 in Groups A, B, and C) while the estrogen module had a weaker correlation with RS. The impact of ER module on RS was stronger in clinical low-risk patients while the effect of the proliferation module was stronger in clinical high-risk patients. The association between the RS and estrogen module was weaker among younger patients, especially in genetic low-risk patients.

**Conclusions:**

RS was primarily driven by the estrogen module regardless of age, but the proliferation module had a stronger impact on RS in younger patients. The impact of modules varied in patients with different genetic and clinical risks.

## Background

Estrogen receptor (ER) is one of the most significant biomarkers of breast cancer, and the ER-positive (ER+) subtype constitutes about 70% of invasive breast cancers (1). Endocrine therapy is essential for all ER+ breast cancer patients, while chemotherapy can improve the prognosis of only a part of this group (2). Several multi-parameter molecular profiling assays were developed to identify ER+ breast cancer patients who can benefit from chemotherapy. The 21-gene recurrence score (RS) is the most widely used assay, which concludes 16 cancer-related genes and 5 reference genes (3). Using fixed coefficients predefined by the regression analysis of gene expression and patient prognosis in the three training studies, patients can be categorized into low-, intermediate-, or high-risk groups. With the results of RS, clinicians can have a clearer understanding about individual patient prognosis and make personalized adjuvant treatment decisions.

Young breast cancer patients (≤40 years old) account for approximately 2%–6% of patient population in RS-related clinical trials (4–6). Previous studies suggested that clinicopathological features in young ER+ breast cancer patients were more aggressive when compared to those in old patients (7–9) and young patients were more likely to benefit from chemotherapy (10). Accordingly, RS was found to show different values when predicting the benefit of chemotherapy in patients of different ages. In the TAILORx trial, researches refined RS groups as low risk (<11), intermediate risk (11-25), or high risk (>25) and discovered that for the majority of patients with RS <25, endocrine therapy alone was noninferior to combined chemo-endocrine therapy. Of note, the interaction between age and RS was significant. For patients <50 years, RS 11-25 might predict some benefit derived from chemotherapy, whereas in patients ≥50 years with a RS 11–25, chemotherapy-derived benefit was absent (11).

The refined ranges of RS can provide more accurate prognosis information and allow certain groups of patients to avoid chemotherapy as well as the side effects along with it. Thus, it is important to understand the biological features as well as molecular drivers behind RS. A previous study discovered that in contrast to the weight of coefficient for calculating RS, the leading molecular driver of RS was actually the estrogen module instead of the proliferation module in the postmenopausal patients (12). However, a similar study in young women was absent. Given the predictive value of RS among different age groups, it is valuable to explore the molecular mechanisms of RS, especially in younger patients.

In this study, we aim to explore the association of RS with its modules and identify the discordance of molecular drivers in patients of different ages.

## Patients and Methods

### Patients

Clinical data of a total of 1,078 unilateral ER-positive and human epidermal growth factor receptor 2 (HER2)-negative female breast cancer patients diagnosed between January 2009 and March 2017 was derived from the prospectively-maintained Shanghai Jiao Tong University Breast Cancer Data Base (SJTU-BCDB). The use of data was approved by SJTU-BCDB for clinical research. Patient information would be collected if it met all of the following criteria: (1) ER positivity with ≥1% immunoreactive tumor cell nuclei determined by immunohistochemical (IHC) staining test (13); (2) HER2 negativity defined as IHC score 0, 1+, or 2+ and/or non-amplified HER2 gene on fluorescence *in situ* hybridization (HER2/centromeric probe for chromosome 17 ratio < 2.0 with average HER2 gene copy number <6.0 signals/cell, or average HER2 gene copy number <4.0 signals/cell regardless of the ratio) (14); (3) intact 21-gene test report. Menopause was determined if: (1) prior bilateral oophorectomy; (2) age ≥60 years old; or (3) age <60 years old, amenorrheic for 12 or more months and the follicle-stimulating hormone and estradiol in the postmenopausal range.

### The 21-Gene RS Assay

The 21-gene tests were performed on formalin-fixed, paraffin-embedded tissue. Hematoxylin and eosin-stained slides were deparaffinized into two 10-µm unstained sections using xylene followed by ethanol as we described in our previous study (15). RNA was extracted and purified using the RNeasy FFPE kit (QIAGEN, Hilden, Germany). Gene-specific reverse transcription was conducted using Omniscript RT kit (Qiagen, 205111, Germany). Standardized quantitative reverse transcriptase-polymerase chain reaction (RT-PCR) was performed in 96-well plates with Applied Biosystems (Foster City, CA, USA) 7500 Real-Time PCR system. RT-PCR was carried out with the Omniscript RT kit (Qiagen, Valencia, CA, USA). Expression of each gene was measured in triplicate, and normalized relative to a set of five reference genes.

### Genetic and Clinical Risk Stratification

As defined in the TAILORx trial (11), we categorized patients into genetic high-risk *versus* low-risk with a cutoff RS value of 25. In addition, patients with tumors of (1) ≤3 cm and Grade I; (2) ≤2 cm and Grade II; (2) ≤1 cm and Grade III were classified as clinical low-risk while others were considered clinical high-risk (4, 11).

### Statistical Analysis

Spearman’s rank correlation was performed to analyze the correlation of RS and its modules. The variance of components of RS was studied in Groups A, B, and C. *p*-value < 0.05 was considered to indicate a statistically significant difference. All tests were performed using R Studio version 1.2.5019 based on R version 4.0.3.

## Results

### Baseline Characteristics

According to the 4th International Consensus Conference for Breast Cancer in Young Women (BCY4) international consensus guidelines (16) as well as patients’ menopausal status, we divided patients into three subgroups: (1) Group A, ≤40 years and premenopausal; (2) Group B, >40 years and premenopausal; (3) Group C, postmenopausal. Among 1,078 cases included in this study, 9.0%, 26.3%, and 64.7% fit into Groups A, B, and C, respectively. The median age was 37 (range 27–40), 47 (range 41–56), and 63 (range 45–93), respectively, in the three subgroups. A total of 31.5% patients had luminal-A tumors (17) and the invasive ductal cancer was the most common histology type (86.4%). Approximately half of the patients had grade II tumors. When using the 8th AJCC staging, 67.9% of tumors were pT1 and 93.4% were node-negative. Among all patients, 638 (59.2%) had RS ≤25 and 440 (40.8%) had RS >25. Forty-nine percent *vs*. 50.5% of the patients had a clinical high-risk *vs*. low-risk. All patients received endocrine treatment. More than half (51.2%) of the patients received chemotherapy (72.2%, 54.2%, 47.1% in Groups A, B, and C, respectively). For premenopausal women, 37.1% of patients ≤40 years and 4.0% of patients >40 years received ovarian function suppression. The distribution of clinicopathologic features in each subgroup was summarized in **Table 1**.

**Table 1:** Basic features of HR+/HER2- early breast cancer patients from SJTU-BCDC.

Characteristics	Total (%)	Premenopausal ≤40 years	Premenopausal >40 years	Postmenopausal
	*n* = 1,078	*n* = 97	*n* = 284	*n* = 697
Median age	58 (24–93)	37 (27–40)	47 (41–56)	63 (45–93)
Subtype				
Luminal-A	340 (31.5)	28 (28.8)	101 (35.6)	211 (30.3)
Luminal-B(HER2-)	738 (68.5)	69 (71.2)	183 (64.4)	486 (69.7)
Pathology				
IDC	932 (86.4)	88 (90.7)	243 (85.6)	601 (86.3)
ILC	46 (4.3)	2 (2.1)	13 (4.6)	31 (4.4)
Others	100 (9.3)	7 (7.2)	28 (9.8)	65 (9.3)
Histologic grade				
1	103 (9.6)	8 (8.2)	31 (10.9)	64 (9.2)
2	559 (51.9)	51 (52.6)	161 (56.7)	398 (57.1)
3	224 (20.8)	28 (28.9)	53 (18.7)	143 (20.5)
Undifferentiated	141 (13.1)	10 (10.3)	39 (13.7)	92 (13.2)
pT				
1	732 (67.9)	64 (66.0)	212 (74.6)	456 (65.4)
2	335 (31.1)	29 (29.9)	71 (26.1)	235 (33.7)
3	11 (0.1)	4 (4.1)	1 (0.3)	6 (0.8)
pN				
0	1,007 (93.4)	94 (96.9)	278 (97.9)	635 (91.1)
1	71 (6.6)	3 (3.1)	6 (2.1)	62 (8.9)
RS score				
≤25	638 (59.2)	58 (59.8)	179 (63.0)	401 (57.5)
>25	440 (40.8)	39 (40.2)	105 (37.0)	296 (42.5)
Clinical Risk				
Low	544 (50.5)	42 (43.3)	165 (58.1)	350 (50.2)
High	534 (49.5)	55 (56.7)	119 (41.9)	347 (49.8)
Chemotherapy				
Yes	552 (51.2)	70 (72.2)	154 (54.2)	328 (47.1)
No	526 (48.8)	27 (27.8)	130 (45.8)	369 (52.9)
OFS				
Yes	47 (4.4)	36 (37.1)	11 (4.0)	0 (0)
No	1031 (95.6)	61 (62.9)	273 (96.0)	697 (100)

HR, hormone receptor; HER2, human epidermal growth factor receptor 2; IDC, invasive ductal cancer; ILC, invasive lobular cancer; RS, recurrence score; OFS, ovarian function suppression.

### Correlation Between RS and Individual Modules

**Figure 1:**
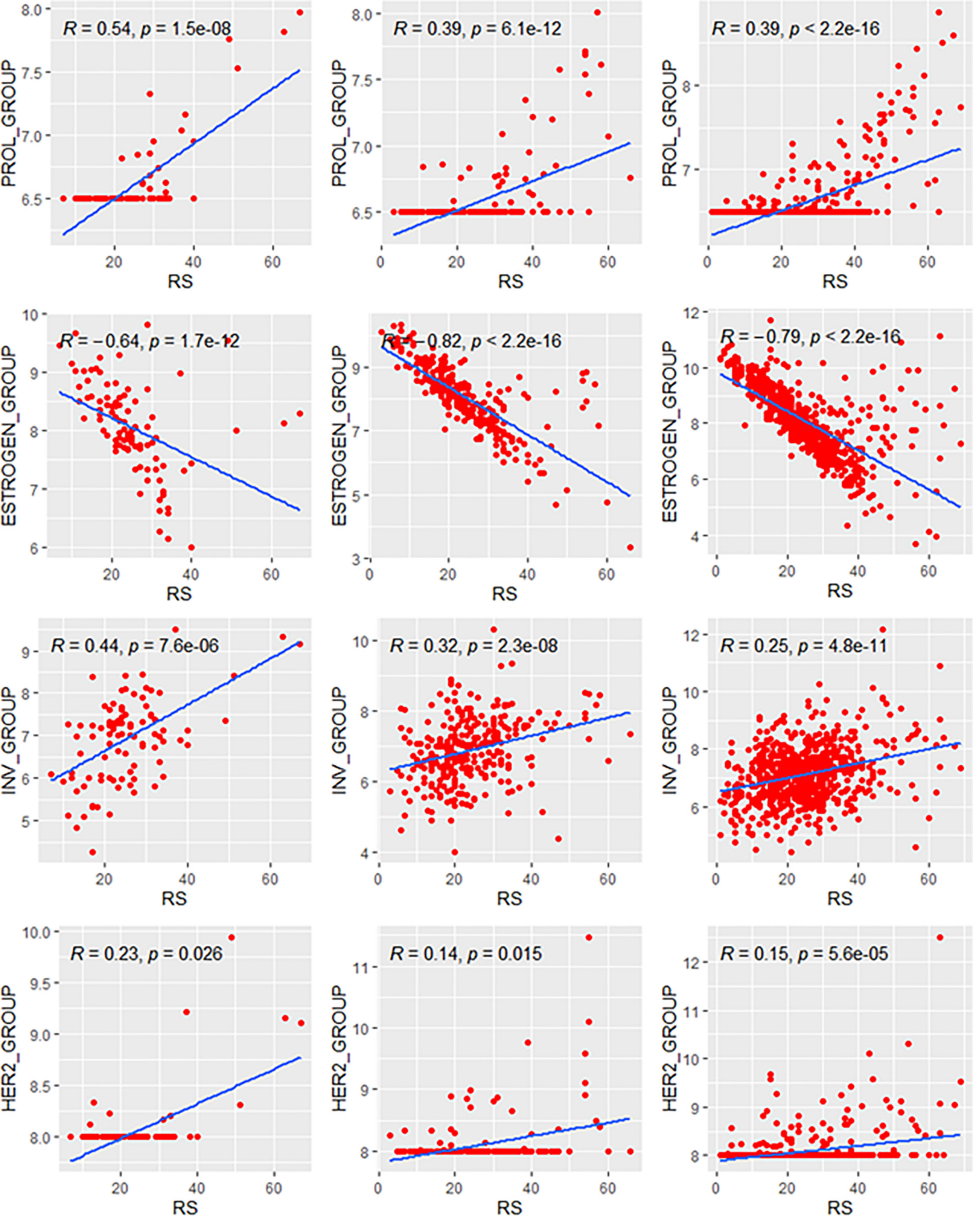
Relationships of the RS with its proliferation module and estrogen module. Groups A, B, C were presented from left to right. RS, recurrence score.

We analyzed the relationship between RS and its constituent modules (**Figure 1**). For the HER2 and proliferation module, the thresholds of 8 and 6.5 were applied. For the estrogen module, it had a stronger negative correlation with RS in patients >40 years (*ρ* = −0.76 and −0.79 in Groups B and C) than in patients ≤40 years (*ρ* = −0.64 in Group A). In contrast, the positive correlation between RS and the invasion module was weaker in patients >40 years (*ρ* = 0.29 and 0.25 in Groups B and C) than in patients ≤40 years (*ρ* = 0.44 in Group A). The coefficients of the HER2 module also showed difference between patients >40 years (*ρ* = 0.14 and 0.15 in Groups B and C) and patients ≤40 years (*ρ* = 0.23 in Group A). For the proliferation module, the impact of RS was similar in premenopausal patients (*ρ* = 0.54 and 0.56 in Group A and B), while it was slightly weaker in postmenopausal patients (*ρ* = 0.39 in Group C). A total of 15.1% patients in our study had the unthresholded proliferation module (19.6%, 12.3%, and 15.6% in Groups A, B, and C).

### Contribution of Individual Modules to the Variance of RS

**Figure 2:**
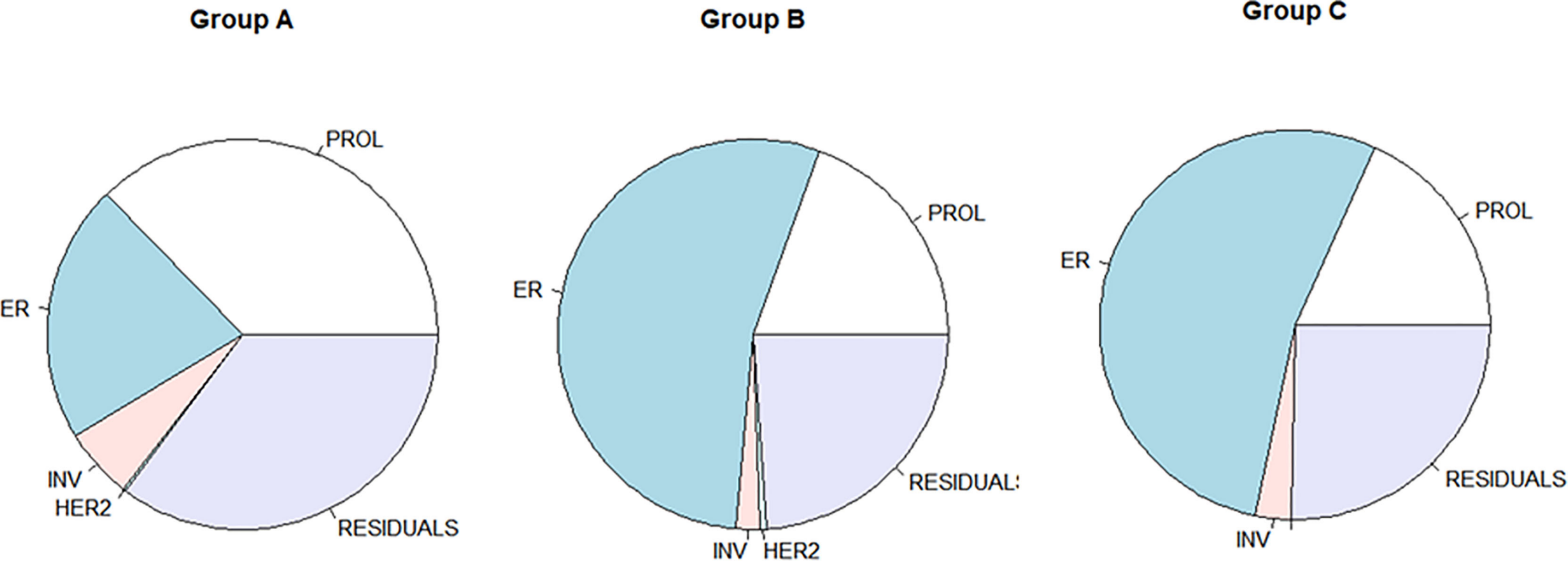
The variance of RS scores as accounted for by individual modules. RS, recurrence score.

The variance analysis was applied to evaluate the ratio of each module contributing to the variance of RS. The distribution of the variance of Groups B and C was similar and showed a different pattern compared with that of Group A (**Figure 2**). In patients <40 years, the variance (37.3% in Group A) of RS mostly derived from the proliferation module. Meanwhile, the estrogen module contributed most variance of RS in the elder patients (54.1% and 53.4% in Groups B and C). In all three groups, the invasion and HER2 module explained little in the variance of RS (shown in **Table 2**).

**Table 2:** The variance of RS as accounted for by individual modules.

RS modules	Group A		Group B		Group C	
	Sum of Squares	Variance Explained (%)	Sum of Squares	Variance Explained (%)	Sum of Squares	Variance Explained (%)
Proliferation (unthresholded)	3,430	37.3	6,125	19.5	16,681	18.2
ER	1,968	21.4	17,025	54.1	48,958	53.4
Invasion	541	5.9	614	2.0	2,779	3.0
HER2 (unthresholded)	24	0.3	170	0.5	81	0
Residuals	3,235	35.2	7,541	23.9	23,113	25.2

RS, recurrence score; ER, hormone receptor; HER2, human epidermal growth factor receptor 2.

### Correlations in Genetic High-Risk and Low-Risk Subgroups

**Figure 3:**
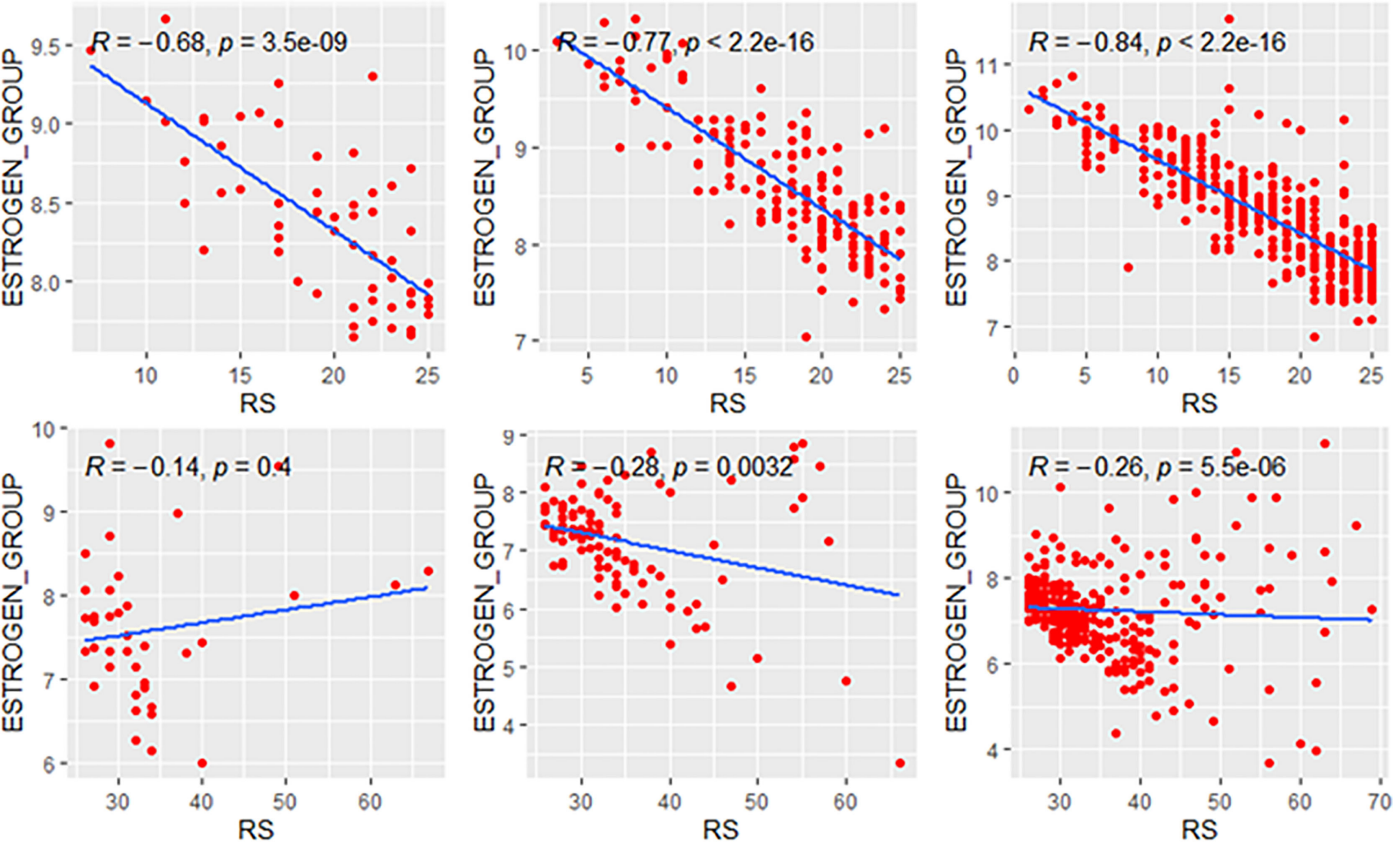
Relationships of the RS with its estrogen module. The upside and downside ranks showed the relationship in patients with RS ≤25 and RS >25, respectively. Groups A, B, and C were presented from left to right. RS, recurrence score.

**Figure 4:**
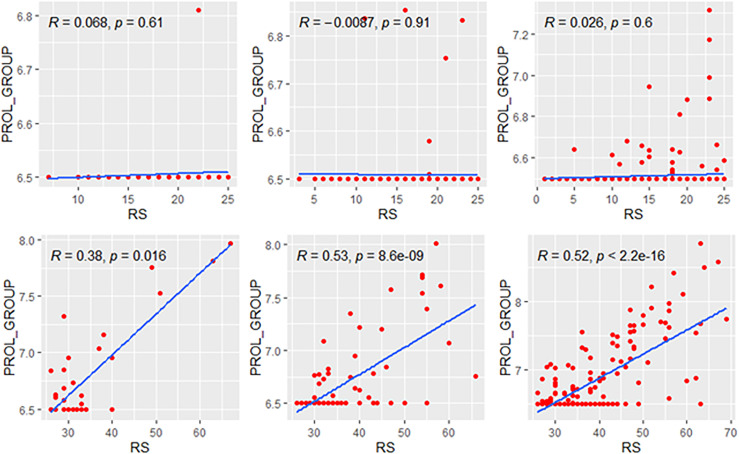
Relationships of the RS with its proliferation module. The upside and downside ranks showed the relationship in patients with RS ≤25 and RS >25, respectively. Groups A, B, and C were presented from left to right. RS, recurrence score; PROL, proliferation.

**Figure 5:**
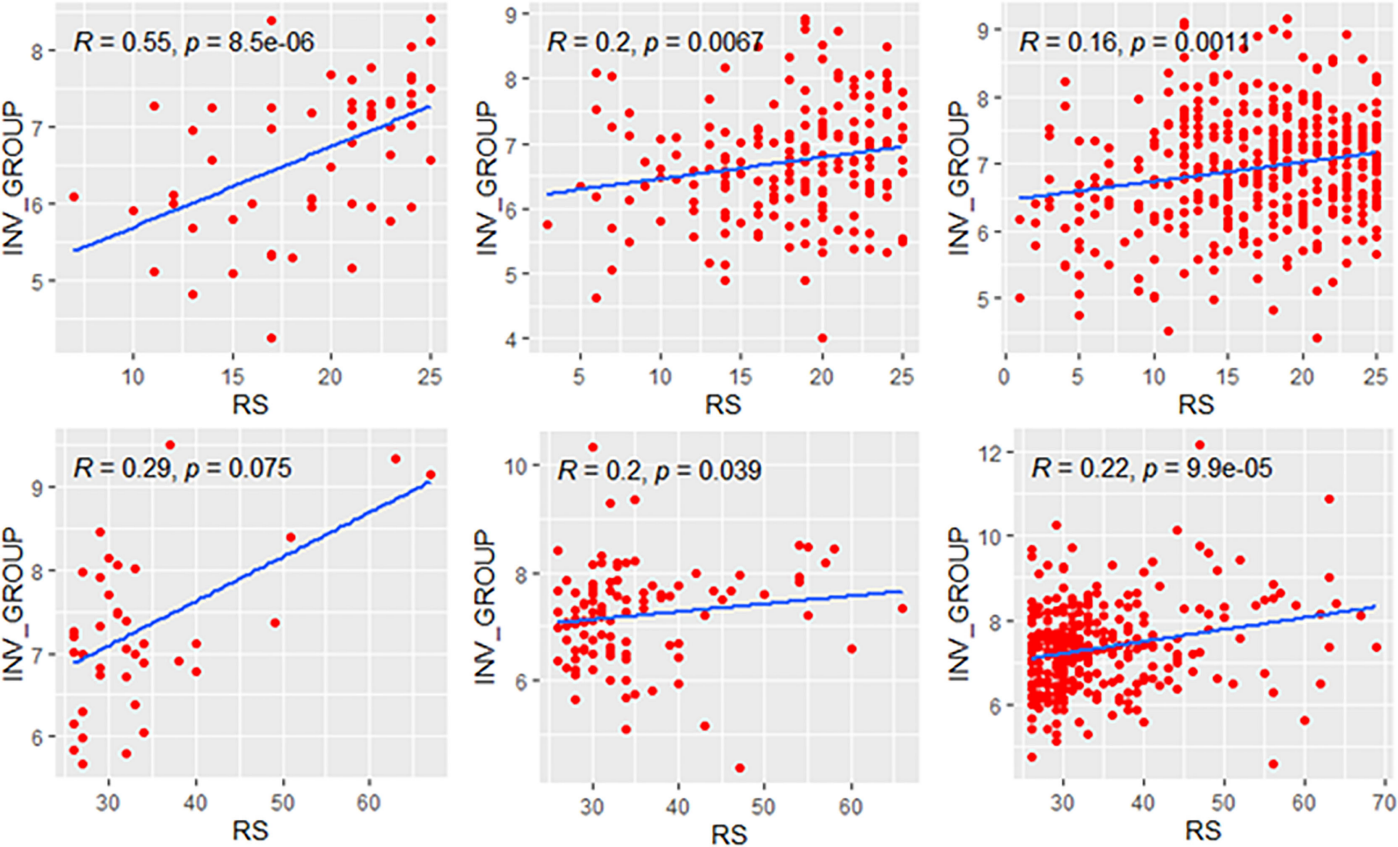
Relationships of the RS with its invasion module. The upside and downside ranks showed the relationship in patients with RS ≤25 and RS >25, respectively. Groups A, B, and C were presented from left to right. RS, recurrence score; INV, invasion.

We explored the correlation of RS with its modules in genetic high-risk and low-risk subgroups (RS>25 and RS ≤ 25, **Figures 3**–**5**). For the estrogen module, its negative impact was much stronger in genetic low-risk patients compared to its high-risk counterparts. Its impact in genetic low-risk subgroup was also stronger in elder patients (*ρ* = −0.68, −0.77, and −0.84 in Groups A, B, and C). For the proliferation module, its positive impact only occurred in genetic high-risk subgroups. Different from the tendency in the whole population (*ρ* = 0.54, 0.56, and 0.39 in Groups A, B, and C), the correlation of the proliferation module with RS reversed between the young and elder patients (*ρ* = 0.38, 0.53, and 0.52 in Groups A, B, and C). For the invasion module, the coefficient was the highest in the genetic low-risk <40-year patients (*ρ* = 0.55) while the difference was not obvious in other patients.

### Correlations in Clinical High-Risk and Low-Risk Subgroups

**Figure 6:**
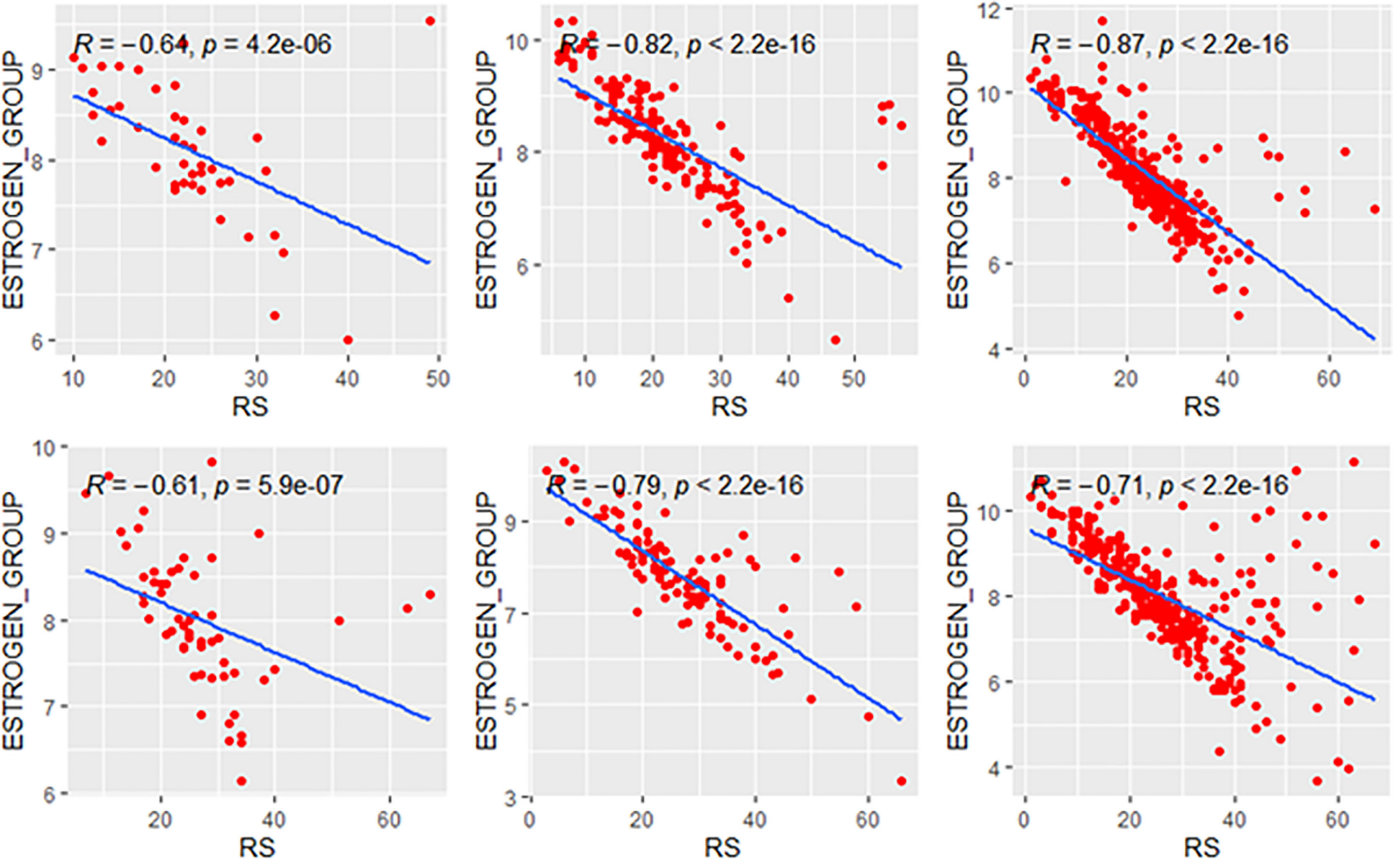
Relationships of the RS with its estrogen module. The upside and downside ranks showed the relationship in patients with low and high clinical risk respectively. Groups A, B, and C were presented from left to right. RS, recurrence score.

**Figure 7:**
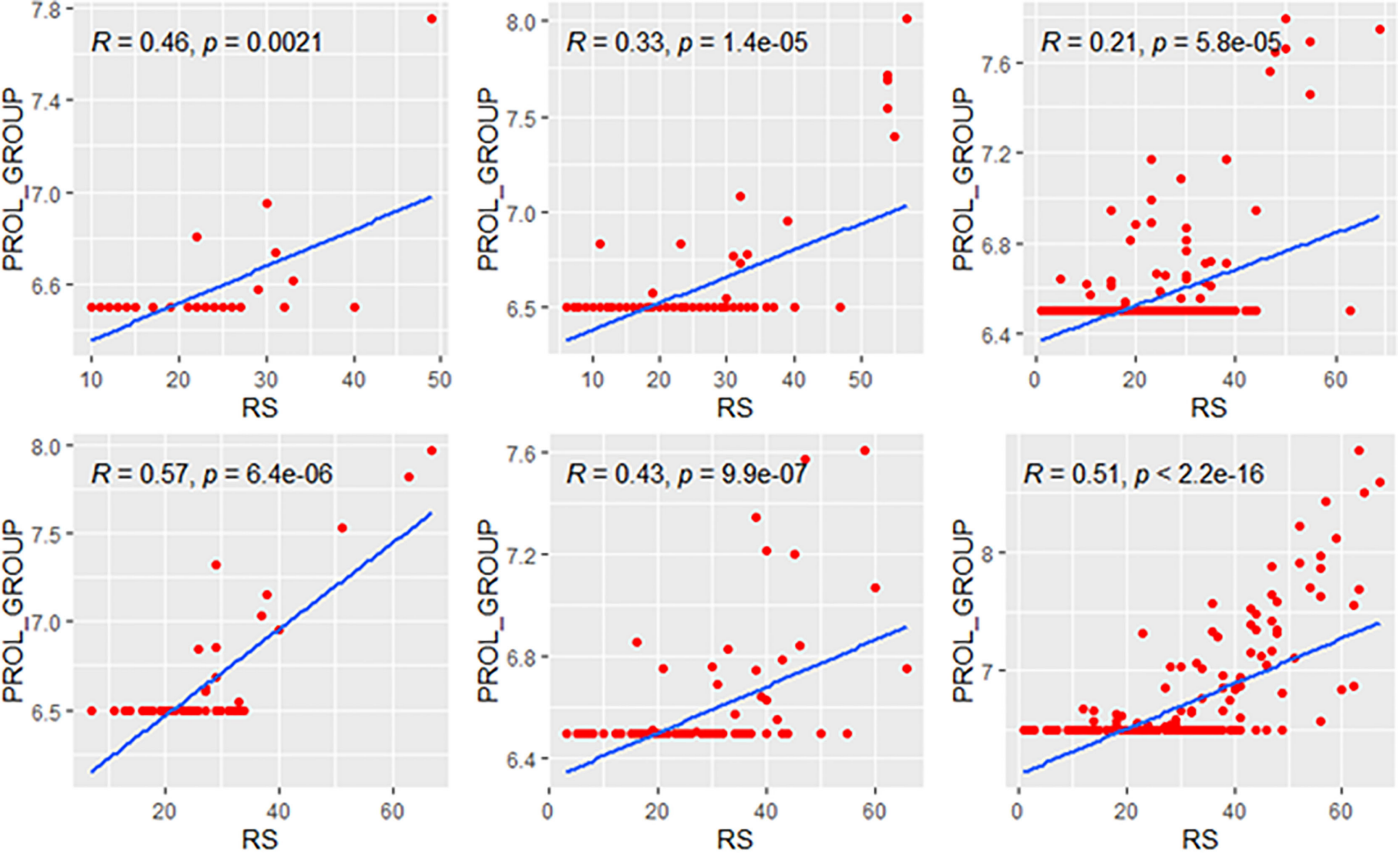
Relationships of the RS with its proliferation module. The upside and downside ranks showed the relationship in patients with low and high clinical risk respectively. Groups A, B, and C were presented from left to right. RS, recurrence score; PROL, proliferation.

**Figure 8:**
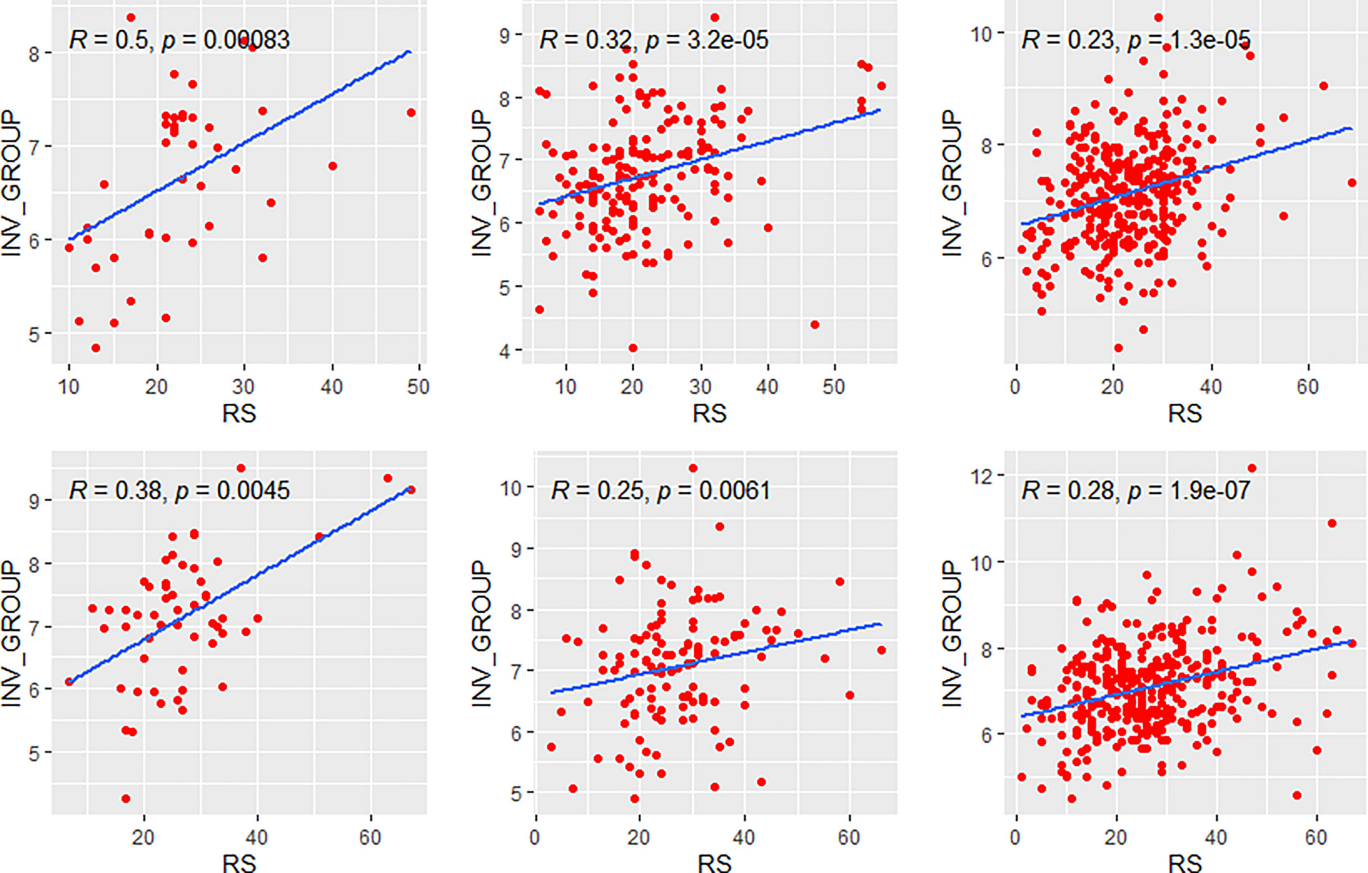
Relationships of the RS with its invasion module. The upside and downside ranks showed the relationship in patients with low and high clinical risk respectively. Groups A, B, and C were presented from left to right. RS, recurrence score; INV, invasion.

**Figure 9:**
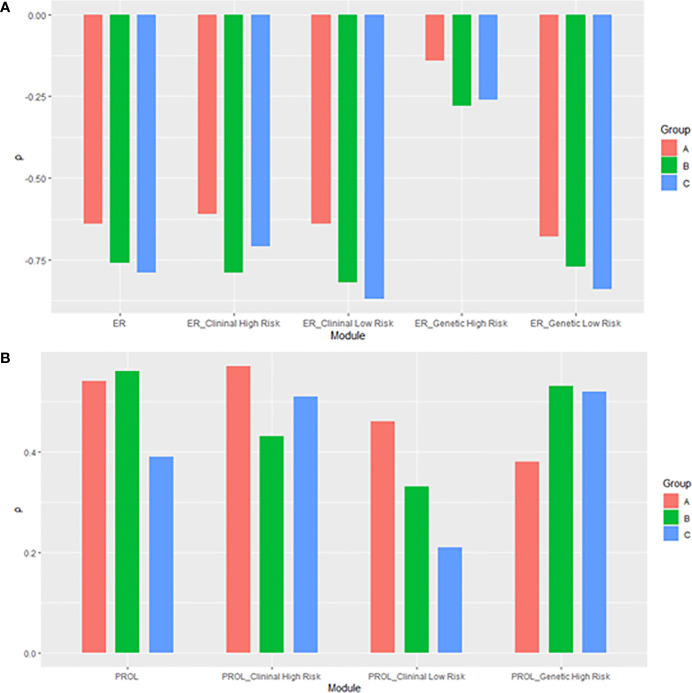
The histogram of relationships of the RS with its **(A)** estrogen and **(B)** proliferation module. The subgroup of proliferation module with genetic low risk was omitted due to non-significance. ER, estrogen receptor; PROL, proliferation.

We further compared the correlations between patients with different clinical risks. The tendency of the correlations between RS and its individual modules was similar between clinical high-risk and low-risk subgroups while some small difference was observed. As for the estrogen module, its negative impact on RS was stronger in patients with low clinical risk compared with high risk (**Figure 6**). For the proliferation module, the positive impact on RS was stronger in high-risk patients regardless of age (**Figure 7**). For the invasion module, the coefficient was stronger in patients ≤40 years old (**Figure 8**). The relationships between RS and its estrogen/proliferation module are summarized in **Figure 9**.

## Discussion

The 21-gene RS was a vital tool to help clinicians predict patient prognostic outcomes and assist treatment decisions. Clinical data showed that patients with the same RS but different ages derived different benefit from adjuvant chemotherapy (11). Thus, it was necessary to understand the internal molecular drivers of RS. A recent study uncovered the discordance of the primary coefficient in the Cox model of RS and the unique molecular features of RS in postmenopausal patients (12). However, data in premenopausal women were insufficient. Here, we made a comparison of the molecular drivers of RS between young and old patients. We found that RS was primarily driven by the estrogen module in patients regardless of age, while the proliferation module had a more substantial impact on RS in patients ≤40 years than in those >40 years.

As reported, patients with the same RS but of different ages might respond differently to the addition of chemotherapy. The result of the TAILORx (11) and the RxPONDER (18) trial suggested that premenopausal patients with RS ≤25 gained a survival improvement from the addition of chemotherapy while the postmenopausal counterparts did not. Likewise, the MINDACT trial (4) showed that for clinical high-risk and genetic low-risk patients, a 5.4% absolute risk reduction of distant metastasis achieved by chemotherapy was observed in patients ≤50 years but not in those >50 years. Based on these results, we divided the patients according to their menopausal status. To explore the mechanisms of RS in patients with different ages, we further categorized patients as young or aged by a cutoff of 40 years old according to BCY4 guidelines.

The results of our study were consistent with the recent study based on patients from the ATAC trial (12). In the ATAC trial, RS was found to be mainly driven by estrogen-related features in postmenopausal women. Our study confirmed that the estrogen module also played a leading role in premenopausal patients >40 years. However, in patients ≤40 years, the link between the estrogen module and RS became weak. Instead, the proliferation module had a strong impact on RS and explained most of RS variance. Given the increased impact of the estrogen module on RS, we assumed that the loss of prediction value of RS after 5 years (19) could be attributed to the strong impact of estrogen module on RS in patients >40 years, because most of them received only 5 years of endocrine therapy. Second, in patients ≤40 years, the weak impact of the estrogen module might be due to relatively lower expressions of ER-related genes. As for the proliferation module, its strong correlation with RS in young patients was in accordance with the previous retrospective studies that young patients were more likely to have tumors with higher grades (9) and higher expression of proliferation related genes (20). In our study, a larger proportion of patients ≤40 years (19.6%) had unthresholded high proliferation module scores than those patients who were >40 years (12.3% and 15.6% in Groups B and C). In fact, the application of threshold distinctly narrowed the gap of proliferation modules’ contribution to RS between patients <40 years and ≥40 years.

In our exploratory analysis, in subgroups with different genetic risks, the association between the RS and its estrogen module was weaker among younger patients, especially in low genetic risk groups. In terms of proliferation-related features, no statistically significant relationship was found between RS and its proliferation module in patients with RS <25, suggesting that proliferation-related features might affect very little in patients with low-to-immediate gene risk. Evidence from TAILORx showed that patients with a mild RS of 11 to 25 could benefit from chemotherapy if they were 41–50 years of age (11). Correspondingly, in our study, RS strongly correlated with the ER module in premenopausal patients who were 40 years or older, while no significant association between RS and the proliferation module was observed. Therefore, a probable presumption was that the chemotherapy benefit for patients 41–50 years old with moderate genetic risk was mainly derived from chemotherapy-induced amenorrhea (CIA), which was common in women 40 years of age or older (21). Over 80% of experts acknowledged the importance of CIA at the 17th St. International Breast Cancer Conference. For these patients, endocrine therapy plus ovarian function suppression might be an alternative option for chemotherapy (22, 23).

Clinicopathological features were traditional important prognostic factors (24). Thus, we investigated the molecular drivers in subgroups with different clinical risks. The negative impact of ER-related features on RS was stronger in clinical low-risk patients. On the other hand, the impact of the proliferation module was stronger in clinical high-risk patients. Our results aligned with previous evidence and suggested that the internal molecular mechanisms might differ even with the same RS. For instance, for a 60-year postmenopausal low clinical risk patient, an RS of 30 might be driven primarily by the strong impact of the estrogen module. Meanwhile, for a similar patient with high clinical risk, an RS of 30 might be attributed to the proliferation-related gene expression. Our results supported the conclusion of the secondary analyses of TAILORx (21). We reconfirmed that clinical-risk stratification (based on tumor size and tumor grade) combined with RS could provide better prognostic information. Additionally, it also explained the better performance of RSClin tool (25) than that of RS alone.

Our study has several strengths. First, we explored the molecular drivers of RS in young patients and compared them with those in elder patients, which had rarely been illuminated before. Second, previous studies were based on samples from the ATAC trial. In the ATAC trial, the majority of patients were clinical low-risk and able to receive tamoxifen or anastrozole alone (26). Instead, patients studied in our study derived from real-world data thus might be more representative of clinical practice. Thirdly, we used a cutoff age of 40 years instead of 50 years to divide customized risk groups. We found distinct patterns of molecular drivers between patients ≤40 years and those >40 years. Thus, it might be necessary to further categorize the ranges of ages in addition to the cutoff of 50 years used by the TAILORx trial and recommended by the ASCO Clinical Practice Guideline (27) and NCCN (28) guideline.

In conclusion, our study confirmed that RS was primarily driven by the estrogen module in patients regardless of age. The proliferation module had a stronger impact on RS in patients ≤40 years than in those >40 years. In RS ≤25 groups, the proliferation module had no apparent association with RS, and thus the chemo-related benefit in young patients might be primarily derived from CIA. In RS >25 groups, the proliferation module became the leading driver, while the estrogen module had a weaker association with RS. The impact of the ER module on RS was stronger in clinical low-risk patients while the effect of the proliferation module was stronger in clinical high-risk patients. Further analysis might pay more attention to the difference between patients ≤40 years and >40 years when using RS to determine the addition of chemotherapy to endocrine therapy.

## Data Availability Statement

The raw data supporting the conclusions of this article will be made available by the authors, without undue reservation.

## Author Contributions

LZ, JW, and MC made the study design. DL, WlC, WgC, and KS participated in data acquisition. JW and MC conducted statistical analysis and manuscript preparation. KS and LZ helped to review the manuscript. All authors contributed to the article and approved the submitted version.

## Conflict of Interest

The authors declare that the research was conducted in the absence of any commercial or financial relationships that could be construed as a potential conflict of interest.

## Publisher’s Note

All claims expressed in this article are solely those of the authors and do not necessarily represent those of their affiliated organizations, or those of the publisher, the editors and the reviewers. Any product that may be evaluated in this article, or claim that may be made by its manufacturer, is not guaranteed or endorsed by the publisher.
